# The association between sleep duration, quality, and nonalcoholic fatty liver disease: A cross-sectional study

**DOI:** 10.1515/med-2023-0670

**Published:** 2023-03-18

**Authors:** Huiwei Liu, Shiliang Huang, Mengdan Xu, Dan Zhao, Xinxue Wang, Liangshun Zhang, Dahua Chen, Jinman Du, Rongbin Yu, Hong Li, Hua Ye

**Affiliations:** Department of Gastroenterology, The Affiliated Lihuili Hospital, Ningbo University, Ningbo, Zhejiang 315040, P.R. China; Department of Gastroenterology, Cixi People’s Hospital, Cixi, Zhejiang 315300, P.R. China; School of Medicine, Ningbo University, Ningbo, Zhejiang 315040, P.R. China; Department of Preventation and Care, The Affiliated Lihuili Hospital, Ningbo University, Ningbo, Zhejiang 315040, P.R. China; Department of Hepatobiliary Surgery, The Affiliated Lihuili Hospital, Ningbo University, Ningbo, Zhejiang, 315040, P.R. China; Physical Examination Center, The Affiliated Lihuili Hospital, Ningbo University, Ningbo, Zhejiang 315040, P.R. China

**Keywords:** Pittsburgh Sleep Quality Index, Munich Chronotype Questionnaire, body mass index, steatosis

## Abstract

Sleep can affect nonalcoholic fatty liver disease (NAFLD). We investigated the association between sleep duration, sleep quality, and NAFLD. From January to December 2018, 1,073 patients (age: 37.94 ± 10.88, Body Mass Index (BMI): 22.85 ± 3.27) were enrolled. Pittsburgh Sleep Quality Index Questionnaire and Munich Chronotype Questionnaire were used to assess sleep duration, quality, and habits. Ultrasonography was used to diagnose NAFLD. Multivariate logistic regression models were used to calculate the odds ratio (OR) and 95% confidence interval (CI) of the risk of NAFLD by different types of sleep duration and sleep quality. No significant differences in sleep time, sleep quality, and sleep habits between the NAFLD and the non-NAFLD groups were observed (*P* > 0.05). There was no correlation between sleep duration and NAFLD in the whole cohort. After adjusting for age, exercise, fasting plasma glucose, and BMI, the group with long sleep duration showed a decreased risk of NAFLD in men (OR = 0.01, 95% CI: 0.001–0.27, *P* = 0.032). However, in all four adjusted models, no correlation between sleep duration, quality, and NAFLD was found in women. In conclusion, sleep duration was significantly and negatively associated with NAFLD in men but not women. Prospective studies are required to confirm this association.

## 1 Introduction

Nonalcoholic fatty liver disease (NAFLD) is defined as the accumulation of triglycerides in the liver of individuals without significant alcohol consumption or viral hepatitis infection [1]. NAFLD has become one of the most common liver diseases worldwide, with a global prevalence of approximately 25% [2]. Unhealthy lifestyle habits, such as excess nutritional intake, less physical activity, or insufficient sleep are thought to be associated with obesity and diabetes. NAFLD is strongly associated with metabolic diseases such as obesity, diabetes, or dyslipidemia which are risk factors for nonalcoholic steatohepatitis, the severe form of NAFLD [3]. There are currently no approved medical therapies for NAFLD [4]. The first-line treatment is lifestyle interventions, such as diet modification and exercise [5,6]. However, compared with nutrition or exercise, sleep improvement has drawn less attention.

Sleep disturbance and deprivation are common medical complaints in modern society [7]. Insufficient sleep may cause metabolic disorders such as insulin sensitivity, obesity, and type 2 diabetes mellitus, and may therefore contribute to the development of NAFLD [8,9]. The associations between sleep duration or quality and the prevalence of NAFLD have been reported in several studies [8–11]. However, the relationships between sleep duration, quality, and NAFLD remain controversial [7–12]. In a community-based cohort study, long sleep duration was associated with the elevation of NAFLD scores in Korean middle-aged adults [12]. In another larger middle-aged Korean population study, short sleep duration and poor sleep quality were significantly associated with an increased risk of NAFLD [10]. A meta-analysis pooled data from six studies and found a small but significantly increased risk of NAFLD among short sleep duration subjects [13].

In the study, we investigated the associations between sleep duration, quality, and NAFLD, as determined by ultrasonography which is a common clinical diagnostic tool for detecting fatty liver [10]. Besides, we examined the gender differences in sleep duration, quality, and their effects on the risk of NAFLD.

## 2 Methods

### 2.1 Study population

**Figure 1:**
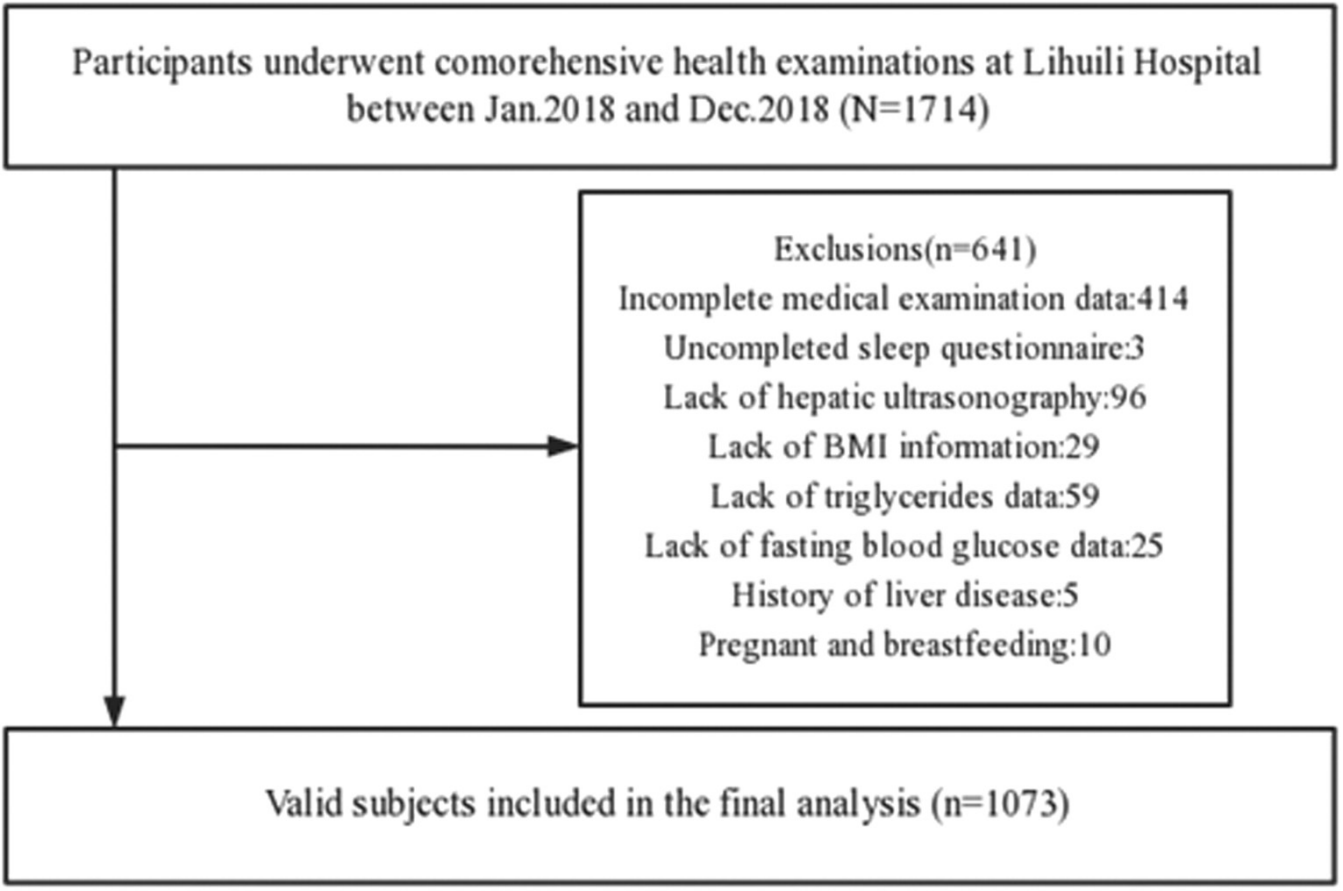
Flowchart of the included subjects.

The subjects in this study were from Lihuili Hospital of Ningbo Medical Center and the Physical Examination Center of Lihuili East Hospital of Ningbo. The study population was restricted to individuals who underwent a health screening examination with information on sleep duration and sleep quality from January 2018 to December 2018 (*N* = 1,714). All subjects received a questionnaire survey and physical examination. The exclusion criteria are incomplete medical examination data, uncompleted sleep questionnaire, lack of hepatic ultrasonography, lack of BMI information, lack of triglycerides data, lack of fasting blood glucose data, history of liver disease, and pregnancy and breastfeeding. A total of 641 subjects met one or more of the exclusion criteria at baseline (Figure 1). The total number of eligible subjects for the study was 1,073. The protocol conformed to the ethical guidelines of the 1975 Declaration of Helsinki and was approved by the ethics committee of the Lihuili Hospital of Ningbo (ethics review number: 2018036). All the subjects knew the purpose of the questionnaire survey and signed the informed consent before entering the study.

### 2.2 Data collection

Sleep duration and quality were assessed using the validated Pittsburgh Sleep Quality Index (PSQI), a self-administered questionnaire. PSQI, comprised of 19 items, generates seven component scores that reflect subjective sleep quality, sleep latency, sleep duration, habitual sleep efficiency, sleep disturbances, use of sleeping medication, and daytime function [14,15]. Poor sleep quality is defined as having a sum of scores greater than 5 for these seven components. Sleep duration was estimated using component 3, which asked for the number of hours of actual night-time sleep during the past month. Sleep duration means the sum of all sleep time in a day. According to the 2015 National Sleep Foundation recommendations for sleep disorders, we classified sleep duration by age group [16]. During health examinations, information such as medical history, medication use, health-related behavior, physical measurements, and serum biochemical measurements is collected. There were questions regarding the amount and frequency of alcohol consumed weekly and daily. We also collected information on smoking history (duration and daily consumption of cigarettes) using questionnaires.

Sleep habits were assessed using the Munich Chronotype Questionnaire (MCTQ) [17]. The assessment of daily life includes the distribution and assessment of daily behaviors, as well as a reminder of working days and rest days [18]. We define 0 as a very early bedtime, 1 as an appropriate early bedtime, 2 as an earlier bedtime, 3 as a normal bedtime, 4 as a slightly late bedtime, 5 as an appropriate late bedtime, and 6 as an extremely late bedtime.

Any type of exercise which lasts longer than 20 min, every time more than 3 times a week, is classified as physical exercise. Depending on the eating habits in the past month, three types of eating habits have been identified: meat-based, vegetable-based, and meat and vegetable equivalent. For Body Mass Index (BMI), we defined BMI < 18.5 kg/m^2^ as lean, 18.5 kg/m^2^ ≤ BMI < 24 kg/m^2^ as normal, 24 kg/m^2^ ≤ BMI < 28 kg/m^2^ as overweight; 28 kg/m^2^ ≤ BMI as obesity. According to the classification by the American Diabetes Association, fasting plasma glucose (FPG) levels were divided into three grades: normal (FPG < 5.6 mmol/L), impaired (5.6 mmol/L ≤ FPG < 7.0 mmol/L), and diabetes mellitus (FPG ≥ 7.0 mmol/L) [19].

To make the questionnaire reliable, the survey was designed according to the basic situation of the Chinese population preliminary design and was discussed, evaluated, and revised by epidemiologists and clinical experts before large-scale application. Before the beginning of the survey, all personnel participating in the survey were given unified training, the questions in the questionnaire were explained one by one, and the relevant controversial issues were standardized. The questionnaire was completed after face-to-face, one-to-one guidance between the investigators and the respondents.

### 2.3 Statistical analysis

The subject characteristics were expressed as mean ± standard deviation for continuous variables or as percentages for categorical variables. The significance of differences in gender, age, smoking, and drinking between NAFLD and non-NAFLD groups was determined by the chi-square test. Continuous variables such as age and BMI were analyzed by a two-sided *t*-test or by analysis of variance. A univariate and multivariate logistic regression model was used to analyze the contributions of different variables. The odds ratio (OR) and 95% confidence interval (95% CI) were used to evaluate the effect of sleep-related variables on NAFLD. The multivariate model was controlled for potential covariates such as age, smoking, drinking, physical exercise, eating habits, and BMI. All statistical analysis was performed by SPSS 18.0 software. All reported *P* values are two-tailed, and the statistically significant threshold was set at 0.05.

## 3 Results

### 3.1 General characteristics of subjects

We enrolled 1,073 valid participants comprising 282 NAFLD and 791 non-NAFLD controls. The demographic and clinical characteristics of the study population are shown in Table 1. The average age of the NAFLD group (41.30 ± 10.04 years) and the non-NAFLD group (36.74 ± 10.92 years) was significantly different (*t* = 6.153, *P* < 0.001). Participants were further divided into six age groups and the differences in age distribution were also significant between NAFLD and non-NAFLD groups (*P* < 0.001). The average BMI of the NAFLD group (25.86 ± 2.76 kg/m^2^) and non-NAFLD group (21.7 ± 2.72 kg/m^2^) was significantly different (*t* = −2.102, *P* = 0.036). The BMI segment distribution was also significantly different (*χ* ^2^ = 319.772, *P* < 0.001). The composition of smokers between NAFLD and non-NAFLD groups was different (*χ* ^2^ = 4.906, *P* = 0.027). The eating habits between the NAFLD group and the non-NAFLD group were also different (*χ* ^2^ = 9.444, *P* = 0.009). The two groups also have significant differences in marital status (*χ* ^2^ = 4.544, *P* = 0.033). However, there was no significant difference in the composition of drinkers (*χ* ^2^ = 0.064, *P* = 0.800) or exercisers (*χ* ^2^ = 2.082, *P* = 0.149) or occupations (*χ* ^2^ = 0.284, *P* = 0.594) between the two groups.

**Table 1:** Comparison of the general demographic of participants

Variables	NAFLD (*n* = 282)		Non-NAFLD (*n* = 791)		*P*
	*N*	Percentage	*N*	Percentage	
Age	41.30 ± 10.04		36.74 ± 10.92		<0.001^a^
					<0.001^b^
<20	0	0	4	0.51	
20–30	34	12.06	241	30.47	
30–40	102	36.17	268	33.88	
40–50	75	26.60	152	19.22	
50–60	64	22.70	108	13.65	
≥60	7	2.47	18	2.27	
Gender					0.001^b^
Men	253	89.72	459	58.03	
Women	29	10.28	332	41.97	
BMI (kg/m^2^)	25.86 ± 2.76		21.7 ± 2.72		0.036^a^
					<0.001^b^
<18.5	0	0	84	10.62	
18.5–24	63	22.34	545	68.90	
24–28	163	57.80	146	18.46	
≥28	56	19.85	16	2.03	
Smoking history					0.027^b^
Smoking now	65	23.05	135	17.07	
No smoking	217	76.95	656	82.93	
Drinking history					0.800^b^
Drinking now	50	17.73	135	17.07	
No drinking	232	82.27	656	82.93	
Eating habits					0.009^b^
Meat	73	25.89	148	18.71	
Vegetable	35	12.41	144	18.20	
Equal	174	61.70	499	63.08	
Physical exercise					0.149^b^
Yes	108	38.30	342	43.24	
No	174	61.70	449	56.76	
Marriage					0.033^b^
Married	231	81.91	599	75.73	
Single^c^	51	18.09	192	24.27	
Occupation					0.594^b^
Yes	265	93.97	736	93.05	
No	17	6.03	55	6.95	

^a^ *t*-Test was used for continuous variables. ^b^Categorical variables were tested by chi-square test. ^c^Being single includes divorce, bereft of one’s spouse, and unmarried. The values in age and BMI rows indicate their average and standard deviation.

### 3.2 Association between sleep duration, quality, habits, and NAFLD

We further examined the associations between sleep duration, sleep quality, sleep habits, and NAFLD in the whole cohort. No significant associations were observed between sleep and NAFLD (Table 2). Multivariate logistic regression analysis for independent variables in NAFLD identified physical exercise as a protective factor (OR: 1.59, 95% CI: 1.11–2.28), while age (OR: 0.97, 95% CI: 0.96–0.99), gender (OR: 0.36, 95% CI: 0.22–0.60), BMI (OR: 0.61, 95% CI: 0.57–0.66), and FPG (OR: 0.63, 95% CI: 0.48–0.81) as risk factors for NAFLD with *P* < 0.05. However, sleep duration, sleep quality, and sleep habits showed no association with NAFLD in the whole cohort (Table 3). To test if the associations exist in different genders, we performed logistic regression analysis for NAFLD and sleep in men and women separately. We found no correlation between sleep habits and NAFLD in both men and women subjects, whether the covariate was adjusted or not. However, we found that the risk of NAFLD was significantly lower in men with recommended or longer sleep duration than in those with too short sleep duration after adjustment for age, exercise, FPG, and BMI (OR = 0.01, 95% CI: 0.001–0.27, *P* = 0.032). No associations between sleep duration, sleep quality, and NAFLD was observed in women (Table 4).

**Table 2:** Summary data for sleep status and subjects composition

	NAFLD		Non-NAFLD		*P*
	*N*	Percentage	*N*	Percentage	
Sleep duration					0.671^a^
Too short	6	2.13	25	3.16	
Recommend	223	79.08	639	80.78	
Appropriate	51	18.09	124	15.68	
Too long	2	0.71	3	0.38	
Sleep quality					0.891^a^
Good (<5)	164	58.16	479	60.46	
Poor (>5)	77	27.30	220	27.81	
PSQI = 5	41	14.54	92	11.63	
Sleep habits					0.951^a^
0 (too early)	6	2.13	22	2.78	
1	13	4.61	38	4.80	
2	43	15.25	133	16.81	
3 (normal)	92	32.62	230	29.08	
4	87	30.85	249	31.48	
5	32	11.35	94	11.88	
6 (too late)	9	3.19	25	3.17	

^a^The categorical variables were tested by chi-square test.

**Table 3:** Multivariate logistic regression analysis for NAFLD

Variables	*B* estimate	Standard error	Wald	*P*	OR (95% CI)
Gender	−1.01	0.25	16.23	<0.001	0.36 (0.22–0.60)
Marriage	0.01	0.23	0.01	0.950	1.02 (0.65–1.59)
Age	−0.03	0.01	8.61	0.003	0.97 (0.96–0.99)
BMI	−0.49	0.04	165.65	<0.001	0.61 (0.57–0.66)
Smoking history	0.08	0.23	0.12	0.732	1.08 (0.69–1.68)
Drinking history	0.33	0.24	1.87	0.171	1.39 (0.87–2.21)
Eating habits	−0.04	0.11	0.33	0.732	0.96 (0.78–1.20)
Physical exercise	0.46	0.18	6.37	0.012	1.59 (1.11–2.28)
FPG^a^	−0.47	0.13	12.40	<0.001	0.63 (0.48–0.81)
Sleep duration	−0.11	0.21	0.26	0.613	0.90 (0.60–1.35)
Sleep quality	−0.01	0.03	0.03	0.871	1.00 (0.93–1.06)
Sleep habits	−0.30	0.07	153.13	0.680	0.97 (0.84–1.12)

^a^Fasting plasma glucose.

**Table 4:** Relationships between sleep duration, sleep quality, and NAFLD

	Univariate analysis OR (95% CI)	Multivariate analysis derived OR (95% CI)			
		Model 1	Model 2	Model 3	Model 4
**Sleep duration**					
Men					
Too short	1.00	1.00	1.00	1.00	1.00
Recommend	0.34 (0.10–1.18)	0.38 (0.11–1.33)	0.37 (0.11–1.30)	0.28 (0.06–1.22)	0.13 (0.02–0.72)
Appropriate	0.30 (0.08–1.10)	0.34 (0.09–1.23)	0.33 (0.09–1.21)	0.25 (0.05–1.12)	0.14 (0.02–0.79)
Too long	0.09 (0.01–1.39)	0.08 (0.01–1.14)	0.08 (0.01–1.16)	0.06 (0.004–1.04)	0.01 (0.001–0.27)
*P*_trend_	0.223	0.221	0.218	0.186	0.032
Women					
Too short	1.00	1.00	1.00	1.00	1.00
Recommend	4.5 (1.13–17.95)	4.32 (0.95–19.71)	3.50 (0.74–16.49)	3.29 (0.70–15.36)	3.60 (0.71–18.25)
Appropriate	2.83 (0.60–13.44)	3.47 (0.63–19.00)	2.40 (0.42–13.73)	2.27 (0.40–12.86)	4.17 (0.61–28.35)
*P*_trend_	0.085	0.165	0.250	0.281	0.276
**Sleep quality**					
Men					
Good	1.00	1.00	1.00	1.00	1.00
Poor	1.08 (0.76–1.55)	1.05 (0.73–1.51)	1.05 (0.73–151)	1.13 (0.78–1.65)	1.15 (0.75–1.79)
*P*_trend_	0.662	0.790	0.795	0.508	0.521
**Women**					
Good	1.00	1.00	1.00	1.00	1.00
Poor	0.59 (0.26–1.34)	0.61 (0.26–1.43)	0.58 (0.25–1.39)	0.59 (0.25–1.40)	0.45 (0.16–1.30)
*P*_trend_	0.205	0.257	0.224	0.231	0.140

Note: Model 1: adjusted for age. Model 2: Model 1 adjusted for physical exercise. Model 3: Model 2 adjusted for FPG. Model 4: Model 3 adjusted for BMI. The too-long sleep duration group for women was deleted as there was only one participant in it.

## 4 Discussion

There are few studies about associations of sleep and NAFLD in Chinese population. In the study, the associations of NAFLD with sleep duration, sleep quality, and sleep habits were evaluated. Sleep duration, sleep quality, and sleep habits showed no statistically significant association with NAFLD in the whole cohort when the model was adjusted for other parameters. It was found that the risk of NAFLD was significantly lower in men with long sleep duration than in those with short sleep duration. However, sleep quality and sleep habits in both genders were not associated with NAFLD.

Previous studies have suggested that sleep quality was associated with NAFLD, and there were sex differences [6]. However, results showed no associations of NAFLD with the global PSQI score, subjective sleep quality score, sleep duration score, and sleep disturbance score [6]. A recent study also showed that a mean sleep time of 7 h or more had a significant negative relationship with NAFLD [20]. Short sleep duration was associated with an increased risk of prevalent NAFLD in Chinese, two South Korean, and American populations [8–11]. Different results were also reported in some South Korean populations. Poor sleep quality but not sleep duration was associated with a lower risk of NAFLD in men. In women, the association of sleep quality and duration with the risk of NAFLD was insignificant [7]. A relationship between long sleep duration and the elevation of NAFLD scores was found after adjusting for several confounding factors in Korean middle-aged adults [12]. Thus, these findings are controversial, although a recent study pooled six datasets and meta-analysis demonstrated a small but significantly increased risk of NAFLD among participants who had short sleep duration [13].

Many reasons may account for the above phenomenon. NAFLD is the most common chronic liver disease worldwide. The disease is a heterogeneous group of liver diseases characterized by the accumulation of fat in the liver [21]. The pathogenesis of NAFLD is not yet fully understood. Many factors may contribute to NAFLD, including diet, medications, genetic predisposition, and gut microbiota [22]. Although this study had a large enough sample size to conclude, it had some limitations. First, the PSQI assessment is a self-reported questionnaire and is subjective. Thus, the associations between sleep quality and NAFLD need to be confirmed by objective methods, such as polysomnography. Second, the study was cross-sectional. The collected data reflect the recent sleep characteristics as well as NAFLD status. Therefore, longitudinal evaluation is essential to reveal associations between sleep quality, duration, and NAFLD in the future. Third, the influences of nutritional factors, genetic background, and gut microbiota are hard to control in the study [22]. Hypercaloric nutrition, including the effects of saturated fat and fructose, as well as adipose tissue dysfunction and intestinal dysbiosis, may contribute to the incidence of NAFLD [23]. In the future study, these factors can be recorded and incorporated into the disease model. Finally, more large-scale cohorts are needed to draw an explicit conclusion. The multi-center study can be carried out to increase statistical power for associations between sleep and NAFLD.

Our result suggests that men with short sleep duration should be cautious about their sleep habits as they have a higher probability to have NAFLD. It has been reported that individuals with NAFLD were more likely to be men and had a higher prevalence of sleep disorders [24]. Thus, the differences in gender should be considered when designing large-scale epidemiologic studies. The molecular mechanisms of chronic sleep deprivation have been extensively studied. Transcriptome analysis identified genes affected by insufficient sleep were associated with circadian rhythms, sleep homeostasis, oxidative stress, and metabolism [25]. Short sleep may increase diabetes risk through three pathways that are alterations in glucose metabolism, upregulation of appetite, and decreased energy expenditure. Type 2 diabetes mellitus is a risk factor often linked with NAFLD [26].

## 5 Conclusions

According to the study, we found that sleep duration is an independent influencing factor of NAFLD in men, and the risk of NAFLD decreases with the increase in sleep duration, but there are no significant associations in women. Thus, men with short sleep duration should be cautious about their sleep habits as they have a higher probability to have NAFLD.
